# Progress of Measles and Rubella Surveillance in the Context of Measles Elimination in the WHO Eastern Mediterranean Region, 2019–2022

**DOI:** 10.3390/vaccines12121349

**Published:** 2024-11-29

**Authors:** Muhammad Farid, Kamal Fahmy, Amany Ghoniem, Md Sharifuzzaman, Quamrul Hasan, Natasha Crowcroft, Patrick O’Connor

**Affiliations:** 1 Immunization Vaccine Preventable Disease and Polio Transition Unit, Department of Communicable Diseases, WHO Regional Office of the Eastern Mediterranean Region, Cairo 11371, Egypt; 2 Department of Immunization, Vaccines and Biologicals, World Health Organization, 1202 Geneva, Switzerlandoconnorp@who.int (P.O.)

**Keywords:** measles and rubella elimination in EMR, measles and rubella surveillance, disease surveillance, epidemiology, disease incidence, immunization coverage, WHO Eastern Mediterranean region and vaccine preventable disease

## Abstract

In 2015, the 62nd session of the Regional Committee [RC] of the Eastern Mediterranean Region [EMR] endorsed the Eastern Mediterranean Vaccine Action Plan 2016–2020 (EMVAP) that included postponement of the measles elimination target to before 2020. However, the EMR does not have a regional rubella control or elimination goal. We reviewed the progress of measles and rubella surveillance in context of measles elimination in the Eastern Mediterranean Region during 2019–2022. We compiled data on coverage, reported cases, surveillance indicators, incidence, and genotypes. We conducted an age-cohort analysis to estimate the size of the susceptible population using coverage and SIAs coverage data. We reviewed the dossiers of countries that applied to the Regional Verification Commission [RVC] for the verification of measles and rubella elimination. Between 2019 and 2022, the regional coverage of the vaccine against measles and rubella was stable at 83% for the first dose [MCV1] and increased from 75% to 78% for the second dose [MCV2] after a dip during COVID-19. In the EMRO, eighteen countries are using MR (measles–rubella) and/or measles–mumps–rubella (MMR) vaccines and four are using measles vaccines. The reported regional measles incidence per 1,000,000 was 23.3 in 2019, decreased to 7.4 in 2020, and re-increased to 50 in 2022, with two main genotypes–D8 and B3–in circulation. Both genotypes are considered to be actively circulating in eighteen countries, with different circulating variants of each genotype. There were no genotyping data available from four countries. Measles and rubella surveillance indicators deteriorated in the region. The number of susceptible individuals exceeded one birth cohort in nine of the 22 countries. In 2019–2022, Bahrain, Egypt, Iran, and Oman were verified to have eliminated measles and rubella. While four countries eliminated measles and rubella and another five progressed toward measles and rubella elimination, however, immunity gaps and reported incidence increased in eleven countries. Countries approaching elimination need to prepare verification dossiers, while others need to increase their routine coverage, conduct follow-up campaigns, and improve surveillance. Submission of progress reports to the RVC will measure progress toward the goal.

## 1. Research in Context

### 1.1. What Is Already Known about This Topic?

The annual reported regional measles incidence increased from 33.5 per million population in 2013 to 91.2 in 2018, primarily because of large outbreaks in Pakistan, Somalia, and Yemen, before decreasing to 23.3 in 2019.

### 1.2. What Is Added by This Report?

In 2015, the member states of the Eastern Mediterranean Region adopted the postponement of the measles elimination goal, setting up a new timeline for measles elimination by the year 2020. Between 2019 to 2022, four countries were verified for measles and rubella (MR) elimination, and another five have been progressing toward achieving MR elimination. Other countries had large immunity gaps for measles and rubella with surveillance deteriorating for various reasons, including the COVID-19 pandemic.

### 1.3. What Are the Implications for Public Health Practice?

Collaborative efforts are needed to mobilize resources to increase routine measles coverage, implement timely high-quality preventive campaigns, and strengthen measles case-based surveillance.

All countries are encouraged to submit early dossiers to the Regional Verification Commission (RVC) for verification.

## 2. Introduction

In August 2020, in resolution WHA 72/90, the Seventy-Third session of the World Health Assembly endorsed The Immunization Agenda 2030: A Global Strategy to Leave No One Behind (IA2030) [1]. The IA2030 outlines goals for reducing morbidity and mortality from vaccine-preventable diseases throughout the course of life, leaving no one behind by increasing equitable access to vaccines for all children [2]. The IA 2030 agenda has identified measles incidence and measles vaccination coverage as an important tracer of inadequate coverage and gaps in immunization for every country, which may also signal people’s inadequate access to primary health care services [3]. This highlights the importance of having a rigorous measles surveillance program to detect immunity gaps and achieve an adequate uptake of at least 95% coverage with two timely doses of a measles-containing vaccine (MCV) among children. The Global Measles–Rubella Strategic Framework 2021–2030 [4], and the Global Measles Outbreaks Strategic Response Plan 2021–2023 [5] align with the IA2030 to achieve measles and rubella elimination by strengthening surveillance for measles and rubella and using effective elimination standard measles surveillance as a critical indicator of the effectiveness of the immunization program.

Between 2000 and 2022, global efforts to strengthen immunization systems across all countries were accelerated, which increased measles vaccination coverage globally until the time of COVID-19 pandemic, when the progress witnessed a backslide. Among the 194 WHO countries, the number of countries that included a rubella-containing vaccine (RCV) in their immunization schedules increased from 132 (68%) in 2012 to 170 (88%) in 2019, and further increased to 175 (90%) in 2022. Between 2000 and 2021, the estimated global coverage with a first MCV dose (MCV1) increased from 72% in 2000, peaked at 86% in 2019, then decreased to 83% in 2020 and 81% in 2021 during the COVID-19 pandemic, the lowest estimated coverage since 2008. Similarly, the global coverage of the rubella-containing vaccine decreased from 69% in 2019 to 68% in 2020 and 66% in 2021, then slightly increased in 2022 to 68%. While surveillance for measles and rubella remained key to achieving the elimination goals, in 2021, only 47 out of the 135 countries reporting (35%), such information achieved the sensitivity indicator target of two or more non-measles, non-rubella discarded rate cases per 100,000 population, pointing to the underperformance and inadequacies of the surveillance system monitoring measles and rubella in many countries. Globally, annual reported measles rates dropped by 88% from 145 to 18 cases per million population during the period 2000–2016, rebounded to 120 in 2019, then declined to 21 in 2020 and 17 in 2021, then increased to 29 in 2022. Between 2000 and 2021, the annual number of estimated measles deaths decreased by 83%, from 761,000 to 128,000 [3], but then increased to 136,000 in 2022 [6]. Globally, reported rubella cases have decreased from 49,180 in 2019 to 17,082 in 2022.

In 2015, the 62nd session of the Regional Committee [RC] of the Eastern Mediterranean Region [EMR] endorsed the Eastern Mediterranean Vaccine Action Plan 2016–2020 (EMVAP) [7]. EMVAP included a postponement of the measles elimination target that had been endorsed by the RC in 1997, which stated that elimination should be achieved at the earliest possible date and before 2020. To achieve elimination, the EMVAP proposed four strategies: First, every district must achieve a high population immunity by achieving at least 95% coverage of two doses of a measles-containing vaccine through routine vaccination and supplementary immunization activities (SIAs) whenever needed. Second, all countries must establish high-quality case-based surveillance with the support of proficient laboratories. Third, countries need to prepare for measles outbreaks and create response plans to detect them and respond with optimal measles case management, including dietary supplementation with vitamin A. Fourth, national immunization programs must communicate effectively to engage communities and build public confidence and demand for immunization. Since the endorsement of the elimination target in 1997, the member states have committed to implementing these regional strategies with various levels of quality.

In February 2018, the WHO Eastern Mediterranean Regional Office (EMRO) established a regional committee for the verification of measles and rubella elimination [8]. This system included establishing a national verification committee in every country and a Regional Verification Commission for Measles and Rubella (RVC-MR) to monitor and verify the status of countries’ measles and rubella elimination. EMRO published a series of reports to document progress toward measles elimination. The last one, published in 2024, indicated that the annual regional measles incidence decreased from 29.8 cases per 1 million population in 2019 to 7.4 in 2020, but then increased 68% to 50.0 in 2022 because of the challenges of providing immunization services and conducting surveillance during the COVID-19 pandemic. In recent times, the molecular characterization data of the measles virus, such as on the circulating genotypes and sequence variants, have formed an integral component of measles surveillance. The identification of the genotypes of confirmed measles cases is an important performance indicator for viral surveillance, which is strongly recommended in support of achieving the national and global goals of measles and rubella elimination. The accurate description of the distribution of measles genotypes in a country or region can provide valuable data on the source of the transmission pathway of the virus, detect imported cases, and can provide evidence of the interruption of the endemic transmission of measles in any country or region. Such data can therefore be used to verify elimination. Conversely, documenting any change in the circulating genotypes can also serve as a valuable tool for measuring the effectiveness of a measles control and elimination program in the countries of the WHO Eastern Mediterranean Region.

By integrating measles genomic surveillance into the epidemiological surveillance of measles, the countries of the WHO Eastern Mediterranean Region can track the circulating genotypes and document the progress of programs to eliminate the transmission of the endemic virus [9,10].

To achieve measles elimination, the report recommended efforts to (a) improve surveillance including laboratory component especially genotyping, (b) increase MCV1 and MCV2 coverage and conduct high-quality supplementary immunization activities, and (c) reach populations at high risk of not accessing immunization services, including those living in areas of civil strife. This report describes the progress of measles and rubella surveillance in the context of measles elimination in the WHO Eastern Mediterranean Region during 2019–2022. The WHO Eastern Mediterranean comprises 21 member states (Afghanistan, Bahrain, Djibouti, Egypt, Iran, Iraq, Jordan, Kuwait, Lebanon, Libya, Morocco, Oman, Pakistan, Qatar, Saudi Arabia, Somalia, Sudan, Syria, Tunisia, the United Arab Emirates, and Yemen) and the occupied Palestinian territory (including East Jerusalem), with a population of nearly 745 million people.

## 3. Methods

We extracted and reviewed the WHO-UNICEF vaccination coverage estimates for the first and second doses of MCV for all of the 21 countries and the occupied Palestinian territory (including East Jerusalem) of the WHO Eastern Mediterranean Region that were available at the time of this analysis, up to the year 2022. These data were shared by the countries with WHO and UNICEF using the Joint Reporting Form. We estimated the coverage using both the routine and supplementary immunization activities carried out in the 21 countries and the occupied Palestinian territory of the WHO Eastern Mediterranean Region from 2019 to 2022. We also reviewed measles surveillance performance and analyzed the epidemiological trends of measles as reported in the case-based surveillance database. Additionally, we reviewed the laboratory surveillance data, including information on the genotyping of the measles virus, from the measles and rubella laboratory network of the WHO Eastern Mediterranean Region, which is a part of the WHO’s global measles and rubella laboratory network.

### 3.1. Immunization

As of 2022, all of the Member States in the region have a routine immunization schedule that include two doses of a measles-containing vaccine, eighteen countries are providing rubella-containing vaccines with measles vaccines. and four countries (Afghanistan, Djibouti, Somalia, and Sudan) are providing only measles-containing vaccines. The WHO’s and UNICEF’s national coverage estimates review the data that WHO and UNICEF receive from all member states through the Joint Reporting Form [JRF] [11], which is submitted to WHO and the United Nations Children’s Fund (UNICEF) on a yearly basis, and available survey results to generate annual estimates of vaccination coverage. Vaccinations against measles and rubella are offered free in the region, and the private sector’s participation in delivering routine immunization services is very limited or marginal.

### 3.2. Immunity Profile

Based on data related to routine immunization coverage including age at administration, SIAs type, age group targeted, and coverage data as well as the anticipated efficacy of the vaccine by the age of administration (85% at 9 months, 95% at 12 months, and 97% at 18 months), we used an age-cohort analysis to estimate the proportion of children remaining susceptible for measles in each age cohort and expressed it as a ratio of the birth cohort [9].

### 3.3. Surveillance System Performance Indicators

All EMR countries except one have established a case-based measles and rubella surveillance system including laboratory testing of all identified suspected cases. Given the disease burden and field constraints, Somalia has implemented aggregate surveillance with limited laboratory testing. All countries use the definition of fever and rash to identify suspected cases and report measles data monthly using a standardized EMRO reporting template. Countries confirm suspected measles cases based on laboratory findings, epidemiologic linkages, and clinical criteria. Measles and rubella laboratory surveillance is based on the serological confirmation of suspected cases and virological characterization of circulating viruses. If a suspected case turns out to be negative for measles by serology, then it is tested for a confirmation of rubella. Countries report viral sequence data to the WHO global measles nucleotide surveillance database [12]. The EMRO monitors case-based measles and rubella in countries using selected surveillance performance indicators. Those include (1) the annualized rate of non-measles non-rubella discarded cases (target rate equal to or exceeding 2/100,000 population), (2) ≥80% of suspected measles and rubella cases meeting the criteria for timeliness and completeness of investigation, (3) ≥80% of suspected cases with adequate specimens collected and tested in a proficient laboratory, (4) ≥80% IgM results reported to national public health authorities by the laboratory within 4 days, (5) ≥80% of specimens received at a laboratory within 5 days of collection. We used the reported incidence rate of rubella and measles cases per 1,000,000 population, the WUENIC estimated coverage of the two MCV doses [13], and the sensitivity of the surveillance system represented by the annual non-measles non-rubella discarded rate equal to or exceeding 2/100,000 population to classify the countries’ progress toward elimination (i.e., eliminated, approaching elimination, or endemic).

### 3.4. Laboratory Methods

EMRO hosts a measles and rubella laboratory network, which is part of the WHO global measles and rubella laboratory network. Each country hosts a national measles and rubella laboratory, while Pakistan hosts a subregional reference laboratory and Oman and Tunisia host regional reference laboratories (RRLs). All of the 22 national laboratories can perform tests of human blood serum samples for the detection of measles and/or rubella specific Immunoglobulin M (IgM) antibody using an Enzyme Linked Immunosorbent Assay (ELISA) In addition, sixteen of the 22 countries established virus detection by Real Time Polymerase Chain Reaction (RT-PCR) or virus isolation in cell culture using a blood sample. Sequencing and genotyping at national or reference laboratories further characterizes viruses. Countries who only have serology capacity can access RRLs for genotyping and sequencing. All of the national measles/rubella laboratories and the RRLs are subjected to regular WHO accreditation to confirm that they are adequate to perform the laboratory surveillance and document progress toward elimination. The molecular surveillance of the measles and rubella virus epidemiology documents the impact of immunization programs, assesses the sensitivity of the surveillance system to detect imported cases, and measures progress toward elimination goals through the identification of the genotypes of confirmed cases. We described each outbreak in terms of sequence diversity, which tracks multiple transmission pathways and monitors the elimination of virus strains and the interruption of endemic virus circulation.

The members of the EMR measles laboratory network submit most of the measles sequence data generated to the measles and rubella nucleotide surveillance sequence database [12], which is a web-accessible and quality-controlled nucleotide database used by the WHO Measles and Rubella Laboratory Network that allow the comparison of identified viruses with viruses in other countries [11].

### 3.5. Regional Verification

The National Verification Committees (NVCs) of countries approaching elimination submit their initial verification reports for the review of the RVC. The EMRO shares the RVC’s comments with the NVCs to finalize their reports. During RVC meetings, the RVC reviews and discusses the dossiers to decide on the elimination status.

The RVC-MR met four times between 2019 and 2022 (including the initial orientation and establishment). In 2019, six countries submitted applications, and three of them were verified for measles and rubella: Iran, Bahrain, and Oman. In 2022, the three countries verified in 2019 were confirmed for sustained elimination status. One additional country (Egypt) was verified for measles and rubella elimination.

## 4. Results

### 4.1. Immunization

In 2019–2022, the estimated regional MCV1 coverage remained stable at 83%, while the estimated MCV2 coverage increased from 76% to 78%. The number of countries that reached the target of 95% coverage for two doses at the national level remained stable at eight from 2019 to 2022. (Table 1). Eighteen countries of the Eastern Mediterranean Region used an MR vaccine in their routine immunization program.

During 2019–2022, 5 countries (Bahrain, Iran, Morocco, Oman, and Qatar) were able to maintain a high coverage of 99% with both doses of both measles and rubella vaccines, seven countries were able to improve their coverage. In seven countries, coverage decreased from 2019 to 2022, and in Somalia coverage remained stable at 46% for MCV1. (Table 1).

### 4.2. Immunity Profile

The ratio of the estimated proportion of susceptible children under five years old to the birth cohort size ranged from 0.4 in Morocco to 2.5 in Somalia, with nine countries having a ratio equal to or exceeding 1.

Surveillance: The key indicators deteriorated from 2019 to 2022. The number of countries that achieved the target of ≥80% of suspected cases having adequate specimens collected and tested in a proficient laboratory decreased from 21 countries in 2019 to 18 in 2022. The regional reported incidence rates of measles reached 23.3 per million in 2019, decreased to 7.4 per million in 2020, re-increased to 9.6 per million in 2021, then jumped to 50 per million in 2022. During 2019, the regional incidence rate of rubella was 3.2 per million, decreased to 0.7 in 2020, then increased to 1.3 in 2021 and 4 per million in 2022 (Table 2). The number of countries achieving 80% timeliness and completeness of investigations decreased from 7 in 2019 to 6 in 2022. The number of countries with a non-measles non-rubella discarded rate ≥2/100,000 population decreased from 14 in 2019 to 11 in 2022 (which might indicate that the reduction in reported cases in 2020–2021 might be false) and the number of countries with ≥80% IgM results reported to national public health authorities by the laboratory within 4 days decreased from 12 in 2019 to 9 in 2022 (Table 2). The number of countries with ≥80% of specimens received at the laboratory within 5 days of collection decreased from 13 in 2019 to 12 in 2022.

**Figure 1:**
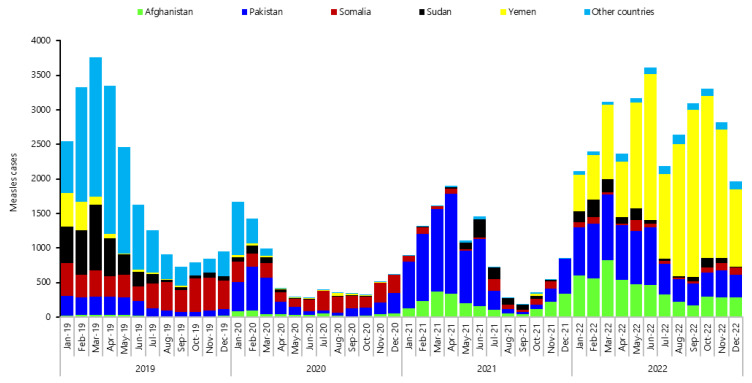
Confirmed measles cases by month of rash onset, WHO Eastern Mediterranean Region, 2019–2022.

**Figure 2:**
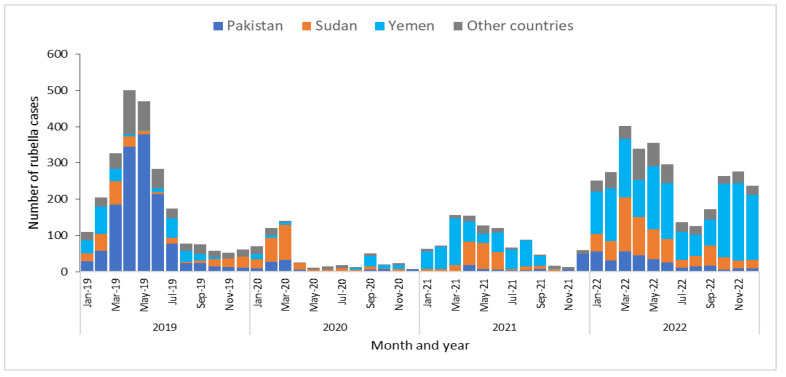
Confirmed rubella cases by month of rash onset, WHO Eastern Mediterranean Region, 2019–2022.

From 2019 to 2022 in the WHO EMR, six countries (Afghanistan, Iraq, Pakistan, Sudan, Somalia, and Yemen) reported high number of measles cases compared to other countries in the region. Measles had two peak seasons during the years 2019 to 2022; one peak appeared from January to April in all years, decreased from May to October, then increased again from November to the end of the year, except for 2022, when cases increased from January to March, decreased in April, then increased in May and June, decreased in July, and increased from August to December (Figure 1). While rubella cases had a peak season from March to May of all years except 2022, when high number of cases was reported from March to May and again in October and November. Rubella cases were reported mainly by Pakistan, Sudan, and Yemen. (Figure 2). There was a drop in reported cases of both measles and rubella during 2020, most probably because of COVID-19.

During 2019, 8 countries reported 95% of the year’s measles cases, compared to 96% of the annual measles cases reported by four countries (Afghanistan, Pakistan, Sudan, and Yemen) in 2022. (Table 1). While 96% of the cases were reported by seven countries in 2019, the majority (57%) of cases were reported from Pakistan, compared to 95% of the cases being reported by six countries in 2022; the majority (53%) of these cases were reported from Yemen.

### 4.3. Genotypes

Table 3 shows that measles genotype B3 and D8 are considered to be actively circulating in eighteen countries, with different circulating variants of both genotypes. The B3 types were more dominant, with 1273 sequences submitted to MeaNS, while the D8 types had 51 sequences submitted. There were no genotyping data available from four countries. During period of 2019 to 2022, rubella genotype 2B was circulating in three countries, (Egypt, Iran, and Oman) of the WHO EMR. Data from other countries on rubella genotyping are not available.

Fifty percent of the circulating B3 sequences were related to thirteen named strains, and most of them related to MVs/Kabul.AFG/20.14/3 (*n* = 231) in 2019–2022, and MVs/Quetta.PAK/44.20 (*n* = 209) 2020–2022, followed by MVs/Kohistan.PAK/51.20 (*n* = 53) 2020–2022, MVs/Bradford.GBR/13.18 (*n* = 44) 2019–2022, MVs/Alburaimi.OMN/15.20 (*n* = 32) 2020–2022, MVi/Harare.ZWE/38.09 (*n* = 21) 2019, MVs/Ohio.USA/37.22 (*n* = 16) 2022, MVs/Salalah.OMN/23.18 (*n* = 10) 2020–2022, MVs/Kansas.USA/1.12 (*n* = 8) 2019–2020, MVs/Islamabad.PAK/1.13 (*n* = 6) 2019, 2021,2022, and MVs/Oslo.NOR/16.18 (*n* = 4) 2019–2022, and two cases reported in 2019 matching MVs/Minnesota. USA/15.17 and MVs/Niger.NGA/8.13.

The remaining 50% of circulating B3 variants (*n* = 637) detected in 2019–2022 are still unnamed.

The reported named strains of the D8 genotype accounted for 86.3% of cases. They were MVs/Gir Somnath.IND/42.16 (20) 2019, MVs/Gaziantep.TUR/13.17 (12) 2019, MVs/Frankfurt Main.DEU/17.11 (10) 2019, 2022, and two cases reported in 2022 matching MVs/Patan.IND/16.19 and MVs/Rudaki.TJK/49.21. The remaining 13.7% were unnamed D8 variants (7) 2019–2022.

## 5. Discussion

The WHO EMR has made substantial progress in reducing measles and rubella cases and deaths since 2000 [8]. The regional MCV1 coverage increased steadily, although it remained below the level of ≥95% necessary to achieve and sustain measles elimination. The WHO Eastern Mediterranean Region, as a whole, did not attain the measles elimination goal. The member states faced challenges, including complex operating environments and the COVID-19 pandemic. Despite the COVID-19 pandemic, NVCs continued to report, the RVC continued to meet and to verify the elimination statuses of the countries in the region, and some progress continued to be made toward measles and rubella elimination.

Eleven Member States of the WHO EMR are in a better situation in the region. Among them, four countries (Bahrain, Egypt, Iran, and Oman) have achieved measles and rubella elimination. Key aspects of the successful member states include sustained high immunization coverage (≥95%) with the two routine doses of measles- and rubella-containing vaccines, supplemented by national or subnational SIAs where required and a strong surveillance system that includes strong laboratory support. This led to zero endemic measles and rubella cases, which was well documented in their dossiers. In these countries, high political commitment and strong health systems were important contributing factors. Seven other countries (Kuwait, Morocco, Palestine, Qatar, Saudi Arabia, Tunisia, and the United Arab Emirates) also have these systems and commitments, but technical issues persist, including gaps in subnational coverage and surveillance indicators that must be addressed.

Unfortunately, the eleven other countries of the region (Afghanistan, Djibouti, Lebanon, Libya, Iraq, Jordan, Pakistan, Somalia, Sudan, Syria, and Yemen) are in crises situations that prevent or hamper system strengthening and the prioritization of elimination. Some of them faced economic crises, armed conflicts, and unpredictable mass population displacements and resettlements. All of these challenges resulted in underperformance of the immunization program and hence measles and/or rubella immunity gaps with a high ratio of susceptible children to the size of one birth cohort. Such immunity gaps lead to an increased risk of frequent large-scale measles and rubella outbreaks that cause a substantial disease burden. Therefore, frequent and/or regular preventive SIAs will be needed in the short term to build immunity and prevent measles mortality. Conducting SIAs in areas with complex humanitarian emergencies requires strong partnerships and local communities’ engagement, the availability of adequate human resources, funds for vaccines and supplies, and the operational costs to achieve high coverage and quality campaigns. In such countries, weak health systems have also prevented the establishment of effective surveillance systems. Moreover, the logistics constraints and limited human resources prevent case identification and investigation, including specimen collection for serology, genotyping, and transport. This is particularly true where the large number of cases increases the need for laboratory supplies and specimen management logistics. In 2020 and 2022, the supply chain disruption secondary to the COVID-19 pandemic led to a global shortage/stockout of measles/rubella laboratory kits. Unfortunately, four countries have not reported measles genotypes for the period 2019–2022. For Djibouti and Kuwait, the program was not collecting clinical specimens. In Morocco, despite collecting many types of specimens for molecular testing, they were unable to genotype. In Yemen, they are collecting some clinical specimens. However, they are not doing genetic analysis or sending specimens to an RRL for genotyping.

Countries that have verified elimination have been able to provide molecular and epidemiological evidence for interrupted measles and rubella viral transmission with no endemic circulation.

From 2019 to 2022, 6 countries (Afghanistan, Iraq, Pakistan, Sudan, Somalia, and Yemen) reported high numbers of cases compared to other countries in the region. Rubella cases were reported mainly by Pakistan, Sudan, and Yemen.

Genotypes B3 and D8 are actively circulating in eighteen countries, with different circulating variants of both genotypes. The B3 types were more dominant, with 1273 sequences submitted to MeaNS, while the D8 types had 51 sequences submitted. There were no genotyping data available from four countries. During period of 2019 to 2022, rubella genotype 2B was circulating in three countries (Egypt, Iran and Oman) of the WHO EMR. Data from other countries on rubella genotyping are not available.

Surveillance activities are challenging, especially in areas with insecure and complex operating environments. As a result, the absence of a strong data system prevents the reporting, analysis, and use of data for action. Poor surveillance leads to delays in notifications of outbreaks and ineffective responses. Many countries have extremely heterogeneous access to immunization and surveillance quality and large populations, and hence also require subnational data to guide public health action tailored to local contexts. In addition, poor data quality of routine immunization coverage and/or campaign coverage assessed by post campaign coverage surveys hampers an accurate assessment of immunity gaps, which makes it challenging to plan timely national campaigns to prevent outbreaks.

Globally, by the end of 2022, no WHO region had achieved and sustained measles and/or rubella elimination [14]. Globally, measles incidence declined during 2020 and 2021, presumably as a result of decreased virus transmission related to COVID-19 mitigation measures and immunity acquired through high rates of infection during the global measles resurgence during 2017–2019 [14]. However, from 2021 to 2022, reported measles cases increased by 67% globally as COVID-19 mitigation measures were lifted and the routine immunization program faced a backslide. We have seen a similar situation in the WHO Eastern Mediterranean Region, where the reported regional incidence rates of measles decreased from 23.3 per million in 2019 to 7.4 per million in 2020 but increased to 9.6 per million in 2021 and 50 per million population in 2022. Similar to the WHO Eastern Mediterranean Region, none of the 47 countries of the WHO Africa region has attained the verification of measles or rubella elimination at the end of 2023, with a total of 125,957 suspected cases of measles being reported through the case-based surveillance system in the countries of WHO Africa region and a regional incidence rate of measles being reported as 60.3 cases per million population, an increase of 22% when compared to the incidence in 2022 (18). Among the other low resourced countries of Africa, the highest national incidence rates of confirmed measles per million population were documented in Liberia (831.6), Gabon (480.4), Equatorial Guinea (268), Central African Republic (240), Cameroon (217.1), and Ethiopia (154.1) [15].

Based on our analysis, we can conclude that the region has experienced heterogeneity concerning progress toward measles and rubella elimination. Eleven of the EMR countries (50%) are doing better, with four countries (Bahrein, Egypt, Iran, and Oman) verified for measles and rubella elimination between 2019 and 2022. The seven countries (Kuwait, Morocco, Palestine, Qatar, Saudi Arabia, Tunisia, and the United Arab Emirates) have coverage, incidence, and surveillance profiles suggesting that they are approaching elimination. The other half of the countries face a widening immunity gap, exposing them to measles and rubella outbreaks. Furthermore, the quality of surveillance is deteriorating in most of the countries in the region. Based on these findings, we can propose three main recommendations. First, countries approaching elimination should prepare and fill dossiers and submit them to the RVC through the NVCs. This will help them understand the requirements for elimination and to practice self-assessment. Second, countries with immunity gaps need to increase routine measles and rubella vaccine coverage and implement preventive campaigns. Third, countries struggling with surveillance performance indicators need to resume efforts, including timely case-based surveillance with complete investigations, laboratory testing, and genotyping so that they can meet key surveillance performance indicators. Along the same lines, countries reporting measles and rubella cases are encouraged to monitor circulating viruses through improved collection of clinical specimens (throat swab, urine, or oral fluid) for virus detection and genotyping. At the regional level, as countries are integrating measles and rubella surveillance, it is time to consider setting a rubella elimination goal to bring the EMR into line with the global MR strategies.

## 6. Conclusions

During 2019–2022, surveillance indicators deteriorated. In 2019, 21 countries achieved the target of ≥80% of suspected cases having adequate specimens collected and tested in a proficient laboratory, compared to eighteen countries in 2022. Fourteen countries achieved ≥2 discarded (non-measles and non-rubella) case rate per 100,000 population in 2019, compared to eleven countries in 2022. The regional reported incidence rate for measles increased from 23.3 per million population in 2019 to 50 per million population in 2022. The regional incidence rate for rubella also increased from 3.2 per million population in 2019 to 4 per million population in 2022. Eighteen countries reported the circulation of the measles genotypes B3 and D8, with different circulating variants of both genotypes. There were no measles genotyping data available from four countries. During the period of 2019 to 2022, rubella genotype 2B was circulating in three countries (Egypt, Iran, and Oman) of the WHO EMR. Data from other countries on rubella genotyping are not available.

All 21 countries and the occupied Palestinian territory of the WHO Eastern Mediterranean Region need to strengthen their measles and rubella surveillance, including obtaining appropriate specimens for genotyping and improving genotyping testing capacity for both measles and rubella.

## Figures and Tables

**Table 1:** MCV1 and MCV2 coverage, number of confirmed measles and rubella cases by country—WHO Eastern Mediterranean Region, 2019–2022.

	2019				2020				2021				2022			
	Coverage (%)		Cases (*n*)		Coverage (%)		Cases (*n*)		Coverage (%)		Cases (*n*)		Coverage (%)		Cases (*n*)	
Country	MCV1	MCV2	No. of Measles Cases	No. of Rubella Cases	MCV 1	MCV2	No. of Measles Cases	No. of Rubella Cases	MCV 1	MCV2	No. of Measles Cases	No. of Rubella Cases	MCV1	MCV2	No. of Measles Cases	No. of Rubella Cases
Afghanistan ***	64	41	212	59	66	43	452	12	63	44	2916	43	68	49	5090	197
Bahrain	99	99	-	-	99	99	-	-	99	99	-	-	99	99	-	-
Djibouti ***	83	81	NR	NR	62	60	NR	NR	50	48	NR	NR	50	48	182	21
Egypt	95	94	-	-	94	94	-	-	96	96	-	-	96	96	-	5
Iran	99	98	-	16	99	98	1	2	99	98	104	28	99	98	-	90
Iraq	82	86	1177	19	76	94	312	-	75	83	15	3	88	97	36	27
Jordan	87	93	45	2	76	90	-	-	76	90	2	-	76	90	21	1
Kuwait	97	94	12	2	95	94	-	-	94	94	4	1	99	94	7	-
Lebanon	82	63	1046	19	74	64	15	-	67	59	5	-	67	59	86	2
Libya	73	72	188	31	73	72	20	3	73	72	5	9	73	72	13	32
Morocco	99	99	12	8	99	99	5	3	99	99	-	1	99	99	2	18
Oman	99	99	-	-	99	99	-	-	99	99	-	-	97	98	-	-
Pakistan	81	74	2066	1370	83	77	2747	99	81	79	7040	98	82	79	7068	309
Palestine	99	99	228	-	99	99	833	-	98	99	-	-	97	93	-	-
Qatar	99	98	5	1	90	88	3	-	99	99	-	1	99	99	4	-
Saudi Arabia	95	96	1035	52	96	96	30	24	98	97	13	4	98	98	149	40
Somalia ***	46	NA ^¶^	4482	-	46	NA ^¶^	2518	-	46	4	746	2	46	8	805	-
Sudan ***	90	74	3555	280	86	68	354	243	81	63	627	251	81	63	1272	694
Syria	65	54	27	4	59	53	15	4	59	53	11	7	41	38	217	23
Tunisia	98	97	4669	162	98	96	11	26	95	98	2	5	95	98	10	16
United Arab Emirates	99	94	186	102	99	92	50	11	99	96	29	1	98	91	98	4
Yemen	67	46	1162	268	68	46	222	88	71	52	5341	530	73	56	23,924	1654
EMR	83	76	20,107	2395	83	77	7588	515	82	77	16,860	984	83	78	38,984	3133

Source: EMR measles and rubella surveillance database. WHO and United Nations Children’s Fund Estimates of National Immunization Coverage (WUENIC). Color scale: Red color is for lower coverage and green is high coverage. *** Countries providing only measles-containing vaccines in their routine immunization schedule. ^¶^ Dose was not included in the vaccination schedule for that year.

**Table 2:** Key surveillance indicators, WHO Eastern Mediterranean Region, 2019–2022.

Indicator	Target	No. (%) of Countries			
		2019	2020	2021	2022
Surveillance quality					
Timeliness and Completeness of Suspected Measles and Rubella Case Investigation	**≥80%**	7 (32)	5 (23)	3 (14)	6 (27)
Percentage of Specimens Received at the Laboratory within 5 Days of Collection	**≥80%**	13 (59)	13 (59)	12 (55)	12 (55)
Percentage of Suspected Cases with Adequate Specimens Collected and Tested in a Proficient Laboratory	**≥80%**	21 (95)	21 (95)	14 (64)	18 (82)
Percentage of IgM Laboratory Results Reported to National Public Health Authorities within 4 days	**≥80%**	12 (55)	12 (55)	11 (50)	9 (41)
Discarded (Non-Measles and Non-Rubella) Case Rate per 100,000 Population	**≥2**	14 (64)	9 (41)	8 (36)	11 (50)
Number of Countries Eliminated Measles	**22**	3 (14)	3 (14)	3 (14)	4 (18)
Number of Countries Eliminated Rubella	**22**	3 (14)	3 (14)	3 (14)	4 (18)
Measles incidence *	**0**	29.8	7.4	9.6	50
Rubella incidence *	**0**	3.2	0.7	1.3	4.0

IgM = Immunoglobulin M. * Cases per 1 million Population.

**Table 3:** Measles Genotyping, circulating variants in the WHO Eastern Mediterranean Region 2019–2022 *.

Country	2019		2020		2021		2022	
Afghanistan								
Bahrain								
Djibouti **								
Egypt								
Iran								
Iraq								
Jordan								
Kuwait **								
Lebanon								
Libya								
Morocco **								
Palestine								
Oman								
Pakistan								
Qatar								
Saudi Arabia								
Somalia								
Sudan								
Syria								
Tunisia								
United Arab Emirates								
Yemen **								
* Based on global surveillance for measles viruses and data reported to MeaNS, which may be incomplete								
B3			D8					
Genotype B3 and D8 are considered to be actively circulating in 18 countries, with different circulating variants of both genotypes								
** Measles genotype data not available in 4 countries								

## Data Availability

The data presented in this study are available on request from the corresponding author.

## References

[1] Immunization Agenda 2030. https://www.who.int/teams/immunization-vaccines-and-biologicals/strategies/ia2030.

[2] Release of the Global Vaccine Action Plan Review and Lessons Learned Report. https://www.who.int/news/item/10-12-2019-release-of-the-global-vaccine-action-plan-review-and-lessons-learned-report.

[3] Minta A.A., Ferrari M., Antoni S., Portnoy A., Sbarra A., Lambert B., Hauryski S., Hatcher C., Nedelec Y., Datta D. (2022). Progress Toward Regional Measles Elimination—Worldwide, 2000–2021. MMWR Morb. Mortal. Wkly. Rep..

[4] Measles and Rubella Strategic Framework: 2021–2030. https://www.who.int/publications/i/item/measles-and-rubella-strategic-framework-2021-2030.

[5] Measles Outbreaks Strategic Response Plan: 2021–2023: Measles Outbreak Prevention, Preparedness, Response and Recovery. https://apps.who.int/iris/handle/10665/340657.

[6] Measles-World Health Organization Factsheet. https://www.who.int/news-room/fact-sheets/detail/measles.

[7] World Health Organization Regional Office for the Eastern Mediterranean. Eastern Mediterranean Vaccine Action Plan 2016–2020: A Framework for Im-plementation of the Global Vaccine Action Plan. World Health Organization, Regional Office for the Eastern Mediterranean, 2019; p. 28. https://apps.who.int/iris/handle/10665/311578.

[8] Goodson J.L., Teleb N., Ashmony H., Musa N., Ghoniem A., Hassan Q., Waciqi A.S., Mere M.O., Farid M., Mukhtar H.E.A. (2020). Progress Toward Measles Elimination—Eastern Mediterranean Region, 2013–2019. MMWR Morb. Mortal. Wkly. Rep..

[9] Guide to the Documentation and Verification of Measles and Rubella Elimination in the WHO Eastern Mediterranean Region. https://apps.who.int/iris/handle/10665/351519.

[10] Mulders M. Manual for the Laboratory-Based Surveillance of Measles, Rubella, and Congenital Rubella Syndrome—TechNet-21. https://www.technet-21.org/en/hot-topics-items/15272-manual-for-the-laboratory-based-surveillance-of-measles-rubella-and-congenital-rubella-syndrome.

[11] WHO/UNICEF Joint Reporting Process. https://www.who.int/teams/immunization-vaccines-and-biologicals/immunization-analysis-and-insights/global-monitoring/who-unicef-joint-reporting-process.

[12] MeaNS2. https://who-gmrln.org/means2.

[13] WHO/UNICEF Estimates of National Immunization Coverage. https://www.who.int/teams/immunization-vaccines-and-biologicals/immunization-analysis-and-insights/global-monitoring/immunization-coverage/who-unicef-estimates-of-national-immunization-coverage.

[14] Minta A.A., Ferrari M., Antoni S., Portnoy A., Sbarra A., Lambert B., Hatcher C., Hsu C.H., Ho L.L., Steulet C. (2023). Progress Toward Measles Elimination—Worldwide, 2000–2022. MMWR Morb Mortal Wkly Rep..

[15] Masresha B.G., Wiysonge C.S., Katsande R., O’Connor P.M., Lebo E., Perry R.T. (2024). Tracking Measles and Rubella Elimination Progress—World Health Organization African Region, 2022–2023. Vaccines.

